# “Let’s put it this way: you can’t really live without it” - digital technologies in routine palliative care delivery: an explorative qualitative study with patients and their family caregivers in Germany

**DOI:** 10.1186/s12913-024-11150-5

**Published:** 2024-06-03

**Authors:** Susann May, Anne Gehlhaar, Kerstin Stahlhut, Marcel-Alexander Kamp, Martin Heinze, Matthew Allsop, Felix Muehlensiepen

**Affiliations:** 1 Center for Health Services Research, Faculty of Health Sciences Brandenburg, Brandenburg Medical School Theodor Fontane, Seebad 82/83, 15562 Rüdersdorf, Brandenburg, Germany; 2 Department of Oncology and Palliative Medicine, Brandenburg Medical School, Immanuel Klinik Rüdersdorf, 15562 Rüdersdorf, Brandenburg, Germany; 3 Department of Psychiatry and Psychotherapy, Brandenburg Medical School, Immanuel Klinik Rüdersdorf, 15562 Rüdersdorf, Germany; 4 Academic Unit of Palliative Care, Leeds Institute of Health Sciences, University of Leeds, Leeds, LS2 9JT UK; 5 AGEIS, Université Grenoble-Alpes, Grenoble, 38000 France

**Keywords:** Telemedicine, Digital Health, Palliative care, Health Services Research, Qualitative research, User perspectives

## Abstract

**Background:**

Despite ongoing efforts to integrate palliative care into the German healthcare system, challenges persist, particularly in areas where infrastructure does not fully support digital technologies (DT). The increasing importance of digital technology (DT) in palliative care delivery presents both opportunities and challenges.

**Objective:**

This study aimed to explore the perspectives and preferences of palliative care patients and their family caregivers regarding the use of DT in care delivery.

**Methods:**

An exploratory qualitative study was conducted using semi-structured interviews with palliative care patients and their family caregivers across various settings. Participants were selected through gatekeeper-supported purposive sampling. Interviews were analysed using structured qualitative content analysis.

**Results:**

Nineteen interviews were conducted.Three themes emerged: (1) Application of DTs in palliative care; (2) Potential of DTs; (3) Barriers to the use of DTs. Key findings highlighted the preference for real-time communication using DTs that participants are familiar with. Participants reported limited perceived value for digital transformation in the presence of in-person care. The study identified requirements for DT development and use in palliative care, including the need for direct and immediate functionality, efficiency in healthcare professional (HCP) work, and continuous access to services.

**Conclusion:**

The findings highlight a demonstrate the importance of familiarity with DTs and real-time access for patients and their families. While DT can enhance palliative care efficiency and accessibility, its integration must complement, not replace, in-person interaction in palliative care. As DTs continue to grow in scope and use in palliative care, maintaining continued user engagement is essential to optimise their adoption and ensure they benefit patients and their caregivers.

**Supplementary Information:**

The online version contains supplementary material available at 10.1186/s12913-024-11150-5.

## Summary Box

### What is already known on this topic

- In Germany, palliative care faces accessibility challenges, particularly for rural residents and middle-aged patients.
- Digital technologies (DT) are increasingly used in palliative care, offering benefits in communication and efficiency but facing integration challenges.
- While healthcare professionals have positively adopted DT, the preferences and needs of patients and family caregivers are less explored.

### What this study adds

- This exploratory qualitative study provides in-depth insights into the perspectives and preferences of palliative care patients and their family caregivers regarding the use of DTs in palliative care delivery.
- It identifies a preference for synchronous communication through familiar technologies, highlighting the critical balance between leveraging technology for care efficiency and maintaining personal interactions.
- The study outlines specific areas of DT application, potential benefits, and barriers to DT use within the palliative care context.

### How this study might affect research, practice or policy

- The findings emphasize the necessity of designing and implementing DTs in palliative care that are aligned with the direct needs and preferences of patients and their caregivers.
- It underscores the importance of personal interaction in palliative care, suggesting that technologies should complement rather than replace face-to-face care.
- Policymakers and healthcare providers may use this study to guide the development of more effective, patient-centered DT interventions in palliative care, potentially improving access and quality of care in underserved areas.

## Introduction

In Germany, approximately 765,000 people require palliative care annually, whilst medical billing data indicates that approximately 400,000 patients in Germany receive palliative care annually [1, 2]. Particularly underserved groups include middle-aged patients [2] and those living in rural areas [1]. Efforts have been underway to integrate palliative care into the German healthcare system [2], yet establishing adequate care services remains a challenge in both rural areas and regional areas with contracting for services that limit capabilities to provide palliative care [1]. For example, access to palliative care advisory teams, which aid healthcare professionals (HCPs) in hospital wards not specialized in palliative care and often serve as the initial gateway to specialized palliative care services, may be restricted and not universally available across all hospital settings [3]. . In addition, the involvement of general practitioners in primary palliative care varies greatly by region [4–6]. This discrepancy highlights a gap in the availability and distribution of palliative care services, underscoring the need for more robust healthcare strategies, as emphasized in studies revealing structural barriers and the necessity for systemic improvements [7, 8].

In recent decades, digital technology (DT) has been increasing in its application in the delivery of palliative care including its use to overcome spatial and temporal distances [9–11]. Digital health refers to “the systematic application of information and communications technologies, computer science, and data to support informed decision-making by individuals, the health workforce, and health systems, to strengthen resilience to disease and improve health and wellness” [9]. Approaches using digital health for palliative care delivery have included videoconferencing or telephony, electronic health records, telephone or cell phone communication, and online interventions such as educational websites and online courses [11]. Existing evidence suggests positive effects of such digital health interventions in terms of training, information sharing, decision-making, communication, and cost-effectiveness [11]. Particularly in remote regions, digital health technologies have the potential to facilitate information sharing and access to palliative care [9, 11]. Nevertheless, despite the growing literature, the effectiveness of DT remains insufficiently demonstrated [11]. However, a lack of integration and interoperability may lead to low engagement and confidence among HCPs, hampering the comprehensive implementation of technologies into routine palliative care [12, 13]. Comparative analysis with other countries, such as the U.S. and the U.K., reveals similar challenges and opportunities in integrating DT in palliative care, suggesting potential areas for international collaboration and learning [14, 15].

To realise the full potential of DTs in palliative care, their development and implementation must be significantly driven by the needs and preferences of end users such as patients, family caregivers, HCPs, and policymakers [16]. Their role as key stakeholders and adopters of DTs is critical to the uptake and adoption of healthcare technologies [17]. In Germany, the increasing digitalisation of the healthcare system is evident through, for example, the implementation of the Digital Healthcare Act in 2019 [18] and the recent Accelerate the Digitalisation of the Healthcare System Act [19]. However, limited research has been undertaken in Germany to explore the needs and preferences of adopters of DT in palliative care. In an earlier exploration of HCPs’ perspectives, experiences, and preferences towards DT use in routine palliative care delivery [8], we found that DTs (i) are widely used in routine palliative care and are positively adopted by HCPs, (ii) can support essential tasks, including workflow organisation, the delivery of patient-centred care, and communication, and (iii) can help bridge geographical and temporal distances, particularly in outpatient care settings. HCPs perceive DTs as having several benefits that contribute to better coordinated, faster, more flexible, and more efficient palliative care [8]. While HCP engagement has highlighted areas for refining and optimising technologies for palliative care, the critical perspective of patients and family caregivers has not yet been explored. These findings align with international studies that also report high levels of HCP satisfaction with DT in palliative care, yet also echo the global call for increased patient and caregiver involvement in the design and implementation of such technologies [14, 20]. Understanding the perspectives and preferences of those receiving, or supporting those in receipt of, palliative care in Germany can guide the development and use of DTs as part of palliative care delivery. Therefore, this study aimed to explore the views and preferences of patients and their family caregivers on the use of DTs as part of palliative care delivery.

## Methods

### Study design

An exploratory qualitative study using semi-structured interviews with patients receiving palliative care across multiple settings and their family caregivers.

### Participants

Participants were selected using gatekeeper-supported purposive sampling [21], to include a heterogeneous sample reflective of perspectives across settings of palliative care in Germany (i.e., specialised outpatient palliative care, specialised inpatient palliative care, hospice care), to participate in the study. The participant recruitment was supported by LAGO (Landesarbeitsgemeinschaft onkologische Versorgung Brandenbrug e.V.). LAGO is a non-profit association in which oncological institutions and professional groups, self-help organizations and volunteers work together to improve oncological care in the federal state of Brandenburg. Other gatekeepers included physicians, psychologists and researcher working with the Working Group for Palliative Care and Psycho-Oncology at Brandenburg Medical School. The project was carried out in the German state of Brandenburg, a state with 2.5 million inhabitants and a low density of physicians. Due to the relatively sparse population, medical care is a challenge. The federal state of Brandenburg is characterized by the lowest density of contract doctors in Germany [22]. Brandenburg has a unique demographic composition; there is a higher proportion of older residents, with a proportion of the population over 60 years old. This ageing of the population mirrors the general trend in many parts of eastern Germany [23]. Inclusion criteria included being a patient and/or family caregiver of a person receiving palliative or hospice care, having the capacity to take part in a half-hour interview, and willingness to participate in the study. Participants were recruited from healthcare institutions which are clinical partners of the psycho-oncology and palliative care Working Group of the Center for Health System Research at the Brandenburg Medical School, representing both rural and urban areas in the state of Brandenburg. Inclusion criteria, capacity, and willingness were assessed by the treating HCPs (i.e., physicians, coordinators, and nurses). If deemed eligible, potential interviewees were invited to participate in the study. The potential participants were given time (in between visits) to consider participation. If they were interested in participating, their contact details were passed on to the study team. The participants did not receive financial incentives.

### Data collection

Interviews were conducted in the German language by an experienced qualitative researcher with a background in psychology, palliative care, and medical ethics (A.G.) using an open-ended interview guide that was developed to elicit participants’ perspectives on their knowledge, use, and perceived benefits and risks of DT use for palliative care. The interview guide was developed by two health services researchers (S.M., F.M.) and A.G. in an iterative review process. Prior to commencing interviews, the interview guide was tested and refined in two face-to-face pilot interviews with treating HCPs (physician and nurse). No revisions were necessary. The final interview guide included the following topics: Knowledge of DT, DT use, and opportunities and risks of DT in palliative care (Supplemental Material 1). Additional open-ended follow-up questions were also included to prompt further inquiry into participants’ perspectives on interview guide topics. Sociodemographic data were collected for each participant, including age, sex, time in palliative care, setting, profession, and whether the participant was a patient or family caregiver. To reduce the risk of infection during the SARS-Cov2-Pandemic and lessen participant burden, participants were able to choose between a face-to-face or telephone interview. Data collection continued until no substantially new findings emerged, and content saturation was achieved. Saturation was defined as code saturation, indicating no additional issues identified, and meaning saturation, indicating no further dimensions, nuances, or insights could be found [24]. The interviews took place between January and May 2023.

### Data analysis

Interviews were audio-recorded and transcribed verbatim. Qualitative analysis of the interviews was performed iteratively by the study team (S.M., F.M., A.G.) based on Kuckartz’s structured qualitative content analysis [25] using MAXQDA Analytics Pro 2022, Release 22.1.0, Verbi GmbH (Berlin, Germany). Relevant text passages from the interview material were coded according to a deductive-inductive procedure. The deductive approach was based on a study conducted immediately beforehand. This study examined the perspectives of healthcare professionals on DTs in routine palliative care [8]. Codes were grouped into categories that merged into a coding tree, which was then discussed by the members of the study team. At this stage, data collection had already been completed. Two researchers (S.M., F.M.) independently applied the coding tree to the entire material. To ensure traceability, the application of the coding tree was validated by a member check with one interview participant.

### Ethical considerations

The study was approved by the data protection officer and the ethics committee of the Brandenburg Medical School Theodor Fontane, Reference ID: E-03-20201123. All study participants received a study information pack and provided their written informed consent prior to voluntary participation. The recorded interviews were pseudonymised after transcription. The coding list was stored securely with access restricted to the study lead and research team members involved in data analysis. Personal data were anonymised in the transcripts. For the presentation of the results, representative quotes from the interview transcripts were selected, translated forward-backward into English, and included in the manuscript. The manuscript has been compiled in accordance with the Consolidated Criteria for Reporting Qualitative Research (COREQ) (Supplemental Material 2) [26].

## Results

Nineteen interviews with 12 patients and 9 family caregivers were conducted and analysed until theoretical saturation was reached. Two of the 19 interviews were conducted with a patient and their relatives. The mean duration of the interviews was 36 (22–54) minutes. The mean age of participants (*n* = 21) was 68 (range: 50–87) years. Most participants were female (14/21; 66%). Detailed characteristics of study participants are shown in Table 1.

**Table 1:** Detailed characteristics of study participants

ID	Age	Sex	in palliative care since (in months)	Setting	Patient/ family caregiver
Interview 1	56	female	5	outpatient	Family caregiver (daughter)
Interview 2	55	female	4	inpatient	Family caregiver (daughter)
Interview 3	61	female	1	outpatient	Family caregiver (daughter)
Interview 4	76	female	6	outpatient	Family caregiver (wife)
Interview 4	76	male	6	outpatient	Patient
Interview 5	62	female	11	outpatient	Family caregiver (wife)
Interview 6	62	female	1	outpatient	Patient
Interview 7	50	female	4	outpatient	Family caregiver (daughter)
Interview 8	87	male	6	outpatient	Patient
Interview 9	58	male	4	outpatient	Patient
Interview 10	55	female	4	outpatient	Family caregiver (wife)
Interview 11	82	female	1	outpatient	Patient
Interview 12	57	male	18	outpatient	Patient
Interview 13	80	male	6	outpatient	Family caregiver (husband)
Interview 14	84	female	18	outpatient	Patient
Interview 15	67	female	n/a	outpatient	Patient
Interview 16	80	female	20	outpatient	Patient
Interview 17	64	female	6	hospice	Patient
Interview 18	83	male	11	outpatient	Patient
Interview 18	84	female	11	outpatient	Family caregiver (wife)
Interview 19	62	male	7	outpatient	Patient

The analysis generated three themes: 1 Areas of application of DTs in palliative care; 2 Potential of DTs; 3 Barriers to the use of DTs. The abbreviations in the results section are used to denote specific roles of the interviewees and to reference specific parts of the data (FC: This abbreviation stands for “Family Caregiver.” It’s used to identify quotes or data that come from a family member who provides care or support to the patient; P: This stands for “Patient.” It indicates that the information or quotes are directly from individuals receiving care; Pos.: This abbreviation stands for “Position.” It is used in conjunction with a number to specify the position of the quote or data point in the interview transcript or dataset).

### Areas of application of digital technologies in palliative care

Patients and family caregivers reported that interaction with physicians and nurses is primarily on an in person level, due to the pre-scheduling of consultations with physicians and nurses. Technology is used when something unexpected happens, an acute event occurs or to re-confirm information.

*“So, as I said, if there’s something unexpected is happening here [participants’ home], I make a phone call, but if I need a consultation with a doctor, I prefer to do it in person, because I want to see him while he is talking to me.”* (Interview 3, FC, Pos. 23).

Most participants preferred synchronous means of communication, such as landline telephones or smartphones, whereby the term “synchronous” refers to real time These were characterised by their direct and immediate functionality, which enables rapid feedback and promotes a speedy, straightforward means of contact. The telephone is therefore mainly used to quickly clarify questions or to reschedule or cancel appointments. It is not commonly used for discussing health-related problems in detail.

*“If you have any questions, you can also clarify them over the phone. Nurse [Name] is a bit of the team lead. And if an appointment is postponed by us or, as you said, by the caregivers, so to speak, we do that by phone, right.”* (Interview 4, P&FC, Pos. 42).

The landline telephone was preferred by participants as it represents a familiar, routinely used device, and was reported to have high connection quality, especially compared to potential connectivity issues that were experienced when using smartphones.

*“Yeah, that’s why the landline is better, but I mean, she doesn’t have a landline in the car. But usually we always try to call from the office [landline].”* (Interview 9, P, Pos. 239).

Furthermore, functions on landline phones including speed dial keys (in which the numbers of those providing care can be stored and dialled directly) and an answering machine function were valued by participants. Often smartphones were used by patients without access to a landline phone, when participants were on the move, and primarily used in emergencies. The risk of inadequate network coverage and the need to recharge phones were seen as burdensome by participants.

A further synchronous communication technology experienced by a few participants was video consultations, often due to limits in mobility. Where experienced it was rated positively and had often been offered by HCPs, in particular by physicians, and participants had been instructed on the procedure for a video consultation.

**Fig. 1:**
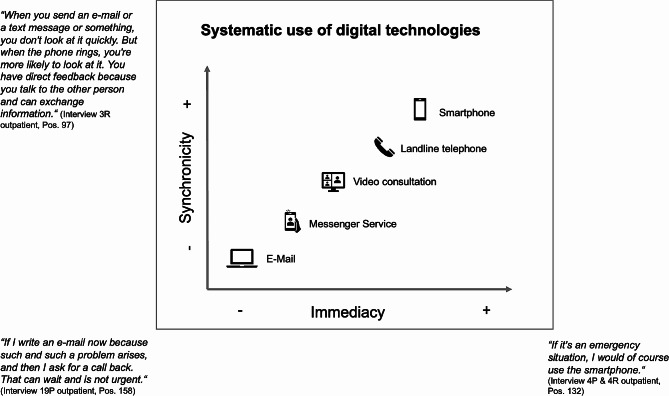
Systematic use of digital technologies in its situational context

Instances of asynchronous communication technologies including messenger services and e-mail were reported, often dependent on individual HCPs. Primarily, these technologies are used if they are explicitly offered by HCPs and a joint agreement is reached on these means of communication. For example, participants reported communication by e-mail is often selected to avoid disrupting daily routines or to send documents, for example medical records. Communication via messenger services is selected when HCPs cannot be reached by phone and concerns do not require direct feedback. The participants often stated that they use messenger services, such as WhatsApp. Communication via messenger services mainly occurs with professionals in specialised outpatient palliative care setting, primarily with nursing staff and volunteers, but in some cases also with physicians. Notably, they are used when the health care institution providing palliative care has no regular opening hours. Figure 1 illustrates the systematic use of DTs in its situational context.

### Routine use of digital technologies

Patients and family caregivers reported that straightforward and low-burden technologies should be used in palliative care, which are already familiar to and used by the users. In addition, the technologies must be efficient in terms of facilitating efficient and rapid communication with HCPs. Above all, patients and family caregivers tend to prefer and feel more comfortable using familiar modes of technology for communication. Reluctance to try out new modes of communication was evident, with a sense that adapting to new modes could be inefficient.

*“I use the landline because I’m quicker and more experienced there. So it’s always a precarious situation when he’s unwell and you’re a bit overzealous. I find it quicker then. Otherwise I use my smartphone.”* (Interview 5, FC, pos. 19).

### Proxy function

Interviewees reported that family members act as a proxy and often take over communication when the patient’s health does not allow them to communicate with HCPs themselves. The role of the family caregiver in providing a proxy function in communication is an important mechanism to ensure that palliative patients can express their needs and wishes for their care.

*“If I need anything, I ask my children and ask them to help me, to get me something or to get me information and so on. So that’s what I try to do through my children.”* (Interview 8, P, Pos. 149).

### Digitalisation - a generational thread

Family caregivers have often raised the issue that older people tend to have a negative attitude towards digital communication tools and are not open to them:

*“As I said, an old person doesn’t look at a cell phone or a laptop like that anymore. For this generation, it’s just no good. Maybe it’s something else for adolescents. But now, for old people like my mom, it’s nothing at all. She doesn’t touch a cell phone, that’s a double Dutch for her.”* (Interview 3, FC, Pos. 117).

Participants reported that older people sometimes have difficulties in dealing with DTs, as they grew up in a time when such innovations did not exist and the rapid change in technology is often a challenge for them. They often lack experience and confidence in DTs, which can lead to a certain scepticism and uncertainty when using digital devices and applications. However, the interviews revealed a predominantly positive attitude on the part of all patient age groups. Irrespective of disease status and health, patient participants had an interest in and openness to the use of DTs in their everyday lives.

*“Yes, it’s just that you can’t get along without it. So why should I close my mind to it?”* (Interview 11, P, Pos. 67).

Some palliative care patients value access to telemedicine services that allow them to receive medical advice remotely, which increases the convenience and flexibility of their care. Overall, palliative patients’ positive attitudes toward DTs demonstrate the potential of eHealth to enrich and improve the lives of people living with challenging health conditions.

*“I think digitalisation in general is helpful and supportive. Nothing works today without it.”* (Interview 11, P, Pos. 187).

Nevertheless, the participants note that humanness must be prioritised when implementing DTs.

*“Digitalisation, yes, I think it’s good, but the human element must not be forgotten.”* (Interview 4, P&FC, Pos. 73).

**Fig. 2:**
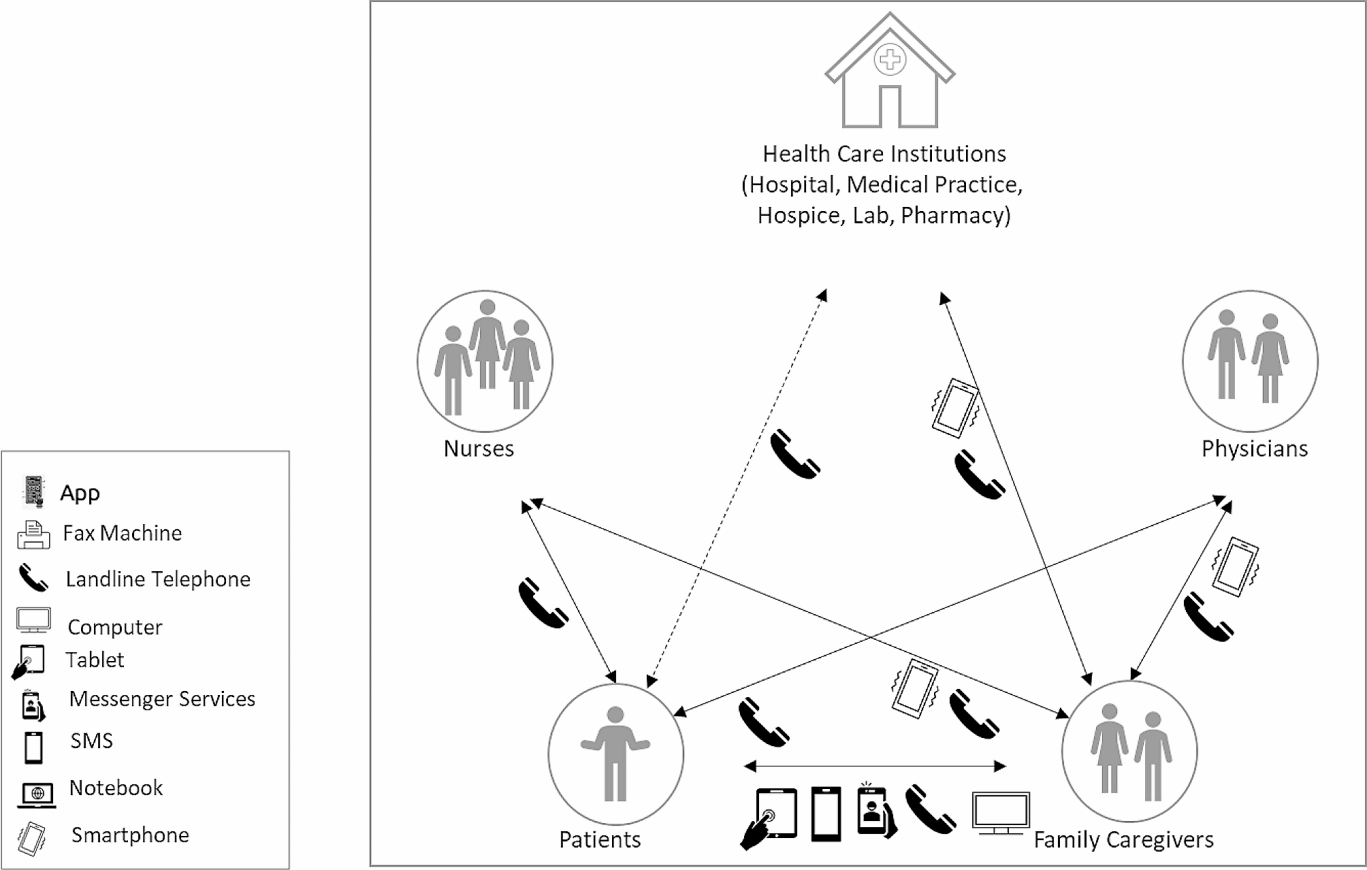
Areas of application of digital technologies from the perspective of patients and their family caregivers

The participants were asked about how different DTs could be used to support the different types of communication in palliative care. The responses of all participants were then systematised. Communication with nursing staff, physicians and other palliative care institutions is usually direct and synchronous via landline telephones or smartphones. In some cases, messenger services are used or emails are written - but these tend to be the exceptions. With regard to communication between patients and relatives, it is not possible to identify any regularity, as all possible devices are used to varying degrees depending on the individual case. Fig. 2 illustrates the areas of application of DTs from the perspective of patients and their family caregivers.

### Potentials of digital technologies

The following three key sub-themes of the potential of DTs were attributed to their preferred use by patients and their family caregivers.

### Support in work processes

Participants reported that DTs can support the work processes of their HCP particularly in the areas of care coordination and information sharing. On the one hand, DTs were seen as a means of enabling HCPs to communicate with each other so that information can be exchanged quickly.

*“Physicians and nurses in particular communicate with each other even more quickly by using technology.”* (Interview 9, P, Pos. 195).

This also was seen as ensuring that patient-relevant information is not lost. Participants acknowledged that DT may reduce the documentation workload for HCPs; for instance, when they use technology for documentation instead of paper-based documentation.

*“So, yes, they don’t have to write long reports anymore by paper, in hard copy, but reports are written right on the spot, which is stored right away with all the other nurses that come in, and doctors and stuff.”* (Interview 1, FC, Pos. 51).

In addition, the participants recognised that time savings may be achieved with DTs. For example, travel distances for patients and physicians can be reduced if communication takes place using DTs. As a result, HCPs have more time for treating patients.

*“These are short distances. And that is an opportunity. You can do all the more for people. If you can handle it in a short conversation then if you first get in the car and have to drive another ten kilometres and then maybe have to call the pharmacy and this and that. You could do all that in a short conversation or in a WhatsApp message or in an email…. you could save yourself the trip and save costs and save time.”* (Interview 6, P, Pos. 111).

### Support in organisation

Participants stated that DTs could contribute to the organisation of appointments.

*“Well, I see more opportunities in appointment management, because if appointments are made and everything goes right, then the person can say, the nurse or the doctor, I can come, there’s something free. So for appointment management, that’s to be welcomed.”* (Interview 5, FC, Pos. 145).

In addition, DTs were recognised as having the potential to enhance and increase the efficiency of processes around organising appointments and medicines management.

*“Yes, I think that some things could be done by e-mail or WhatsApp or something like that, if it would be a relief for the others. Like, for example, reordering medication or something like that.”* (Interview 12, P, Pos. 20).

**Accessibility of care**From the point of view of patients and their family caregivers, the use of DT was recognised as offering numerous advantages, especially for people with limited mobility. By using DT, people with physical limitations can gain access to healthcare services.

*“Because, as I said, I’m not starting from myself now, because I’m still relatively mobile and active. But anyone who is really suffering from pain is given morphine, lies in bed, and perhaps has a gastric tube inside. They’re just trapped in their body and can’t do anything anymore. But if he still has the cell phone or tablet in front of his eyes and can even control a few things with it, whether it’s the TV, whether it’s the radio or maybe communicating with the neighbours or calling for help or whatever, that’s a great thing.”* (Interview 19, P, Pos. 148).

Virtual care was recognised as a means of delivering psychosocial care, providing a safe space where severely ill patients and their family caregivers can open up about their challenges and receive support. Through social media, messaging platforms and online communities, they can maintain their social contacts and make new connections, which might reduce feelings of isolation.

*“If someone is still standing by me a bit, that’s quite good. But for the person who can no longer do that, who is stuck in bed, for example…. and also the doctor or the nurse can’t scurry around him all the time. […] That is not possible. Then the nursing service is something else again than this care. If you can reach this care through social media, I think that’s a great thing.”* (Interview 19, P, Pos. 108–110).

Patient monitoring was a further aspect identified by patients. People with long-term medical needs may require regular check-ups and treatments, which often involve travel. Through the use of DT, participants identified that HCPs could monitor the condition of their patients and intervene when necessary, without having to be physically present.

*“In this respect, I see huge opportunities in the fact that [the use of digital technology] can be expanded to work with it. That it can bring advantages for both, the HCP and for you as a patient, that communication is expanded. It’s not just a matter of a brief ten-minute consultation with the doctor followed by weeks of disconnection. In this scenario, both parties remain largely unaware of each other’s post-consultation experiences. The doctor is left in the dark regarding the patient’s well-being after prescribing medication. With digital technology, this knowledge gap can be bridged through feedback, greatly enhancing the overall healthcare experience. You can give feedback. Feedback. And that’s much better there.”* (Interview 19, P, Pos. 202).

Lastly, DT was recognised as having the potential to enable rapid accessibility and availability of information and services.

*“A: And why should digital technologies be used in palliative care or not?*

*B: Yes, they have to be used because of people… Time is money. And as I said, sometimes it goes with a small short answer to fix a problem. And it doesn’t always have to be a huge effort. If I have access to a competent person, a trained person, at any time, and can exchange a few words with them, then that is important and valuable to me.”* (Interview 6, P, Pos. 54–55).

### Barriers to digital technology use

Barriers to the use of DTs can take various forms and occur at both the communicative, individual, and structural levels .

### Communication restrictions

One of the most prominent barriers to the use of DTs reported by family caregivers was a risk of misunderstandings. Communication taking place via DT may occur without face-to-face physical communication. Emotions, tone of voice, and body language are harder to interpret, which can lead to misinterpretations and conflicts.

*“Yes, there are many interpersonal problems because people write too much to each other and misunderstandings are inevitable.”* (Interview 6, P, item 147).

DT enables communication over long distances but was recognised as often lacking direct feedback in real-time. In a face-to-face conversation, participants felt they could respond immediately to questions or concerns, but when using DTs, there can be delays, which can reduce the efficiency of and create stilted communication.

*“And difficulties, yes, if care were limited to e-mail contact, then I would not be so pleased, because I’m afraid that it would be delayed or lost or something. I always have to hear him* [the doctor] *say, yes, I will, don’t worry, I’ll pass it on or something. If I just write that, then I don’t get feedback right away.”* (Interview 5, FC, Pos. 139).

A further obstacle to the effective use of DTs is the perception of them not as a means to improve care, but as an end in itself. If patients do not understand why certain technologies are being used and what their intended benefits are, they lack the motivation to engage with and use these technologies as part of their care. Additionally, habits and routines have been identified as influential factors, as they can lead to the perception that the use of technology is not an improvement. Patients are so accustomed to traditional communication channels (e.g., landline phones) that they are not open to using new technologies, even if these could increase efficiency.

*“Yes, because the need has not yet arisen. So by smartphone phone, yes, but otherwise I haven’t had to forward any doctor’s reports yet. […] There isn’t much communication between different doctors, SOPC and so on is necessary.”* (Interview 1, FC, Pos. 43).

DT-based communication was often perceived as impersonal. The humanness might be lost because there is no direct personal contact.

*“But I think you also have to be careful not to lose focus on the person. Because you simply see certain things better in person when you have the patient in front of you. But in principle, it’s certainly good because it saves the nurse work and time, but as I said, I think people are simply left out of the equation when it comes to intensive digitisation, and it’s all about or should be about, people.”* (Interview 1, FC, Pos. 17).

In a face-to-face conversation, the conversational partners are physically present and can connect directly with each other. Physical presence creates a human connection and fosters a sense of closeness and familiarity. Digital conversations lack this physical interaction, which can lead to them being perceived as more distant and impersonal.

*“Exactly, that’s the point, because these are also people who can no longer participate in life due to their serious illness, who are confined to their four walls. I would say that when someone comes and pats your arm a bit and says, well, how are you, it’s always very different than when he sits at the screen and says, so now, tell me. I find it very impersonal and detrimental to the clinical picture when it’s done that way.”* (Interview 5, FC, Pos. 173).

### Individual barriers

A common barrier to the effective use of DTs is patient disinterest. Some patients reported that they do not need the technology or that they can manage their tasks without DT.

*“Because I’m not interested in technology at all. What I can do physically, I do; what I can do normally, I do. I am under medical supervision and that is enough for me. And if someone wants to reach me and wants to talk to me, he can reach me at any time via my landline phone. The answering machine is also on, so from there, I have no problems.”* (Interview 18, P&FC, Pos. 59).

Lack of knowledge about available digital options and how to use them is another significant barrier. Participants reported that they are not sufficiently informed or do not have the necessary skills to use DTs effectively.

*“And I see this risk that, in old age, people will no longer be able to do this themselves or to communicate via this channel that we are currently trying. And that is also necessary. And I see this risk.”* (Interview 4, P&FC, Pos. 205).

It was evident that there was wider variation at the individual level in terms of affinity to technology from the patients’ perspective, from enthusiastic to reluctant and uncertain.

### Structural barriers

One of the fundamental structural barriers to the use of DTs is the availability and reliability of network coverage. Inadequate internet connectivity was reported by participants, noted to be particularly problematic in rural or remote areas in Germany. Slow Internet speeds and unstable connections were recognised as having the potential to affect the use of technology and effective communication.

*“That’s out here in the village, too, sometimes really with connection problems, with dead holes and so on.”* (Interview 7, FC, Pos. 161).

The protection of personal data too was a concern for some participants. For example, some participants expressed concern that sensitive, health-related information could be accessed. However, other participants attributed data protection to a subordinate role:

*“It’s all a bit blown out of proportion with data protection. Sure, there are some things you know you can’t talk about in public or whatever. But no, I don’t worry about that so much. Data protection doesn’t play a role.”* (Interview 5, FC, Pos. 163).

### Frugality towards healthcare

The non-use of DTs primarily arises from a lack of recognition of their necessity and frugality. Patients already feel well cared for and demonstrate an understanding of the limited resources and stress faced by HCPs. They consider the workload of HCPs by - if visible to the patients - communicating their concerns asynchronously rather than synchronously, allowing HCPs to respond more flexibly.

*“Then I write an e-mail and either the doctor calls me back or he says I’ll turn up then and there. Then we sort it out, depending on how urgent it is. But I usually write an email first before I intercept the person at work. After all, they have work to do.”* (Interview 6, P, Pos. 63).

If good quality standard care can be accessed, there is little perceived need or value for digital approaches. There was little appetite for digital transformation to care as patients and families were content with the palliative and hospice care they were accessing.

## Discussion

This study identified patient and family caregiver views on and preferences for the use of DTs in palliative care. Key findings include an emphasis and desire for synchronous communication that leverages technologies familiar to patients and family caregivers, with a limited value of digital transformation perceived when there exists high-quality access to quality care. The study elicited key requirements that can be used to guide the development and use of DTs in the context of palliative care delivery in the region in which the research was conducted, as outlined in Table 2. Requirements include current uses of DTs, alongside personal and contextual factors that may influence their use by patients and their family caregivers.

**Table 2:** Summary of patient and caregiver requirements to consider in the development and use of digital technologies for palliative care

Theme	Derived requirements
The use of DTs in palliative care	- Direct and immediate functionality of mobile or landline calls are preferred and enable rapid feedback and promote a speedy, straightforward means of contacting HCPs - Asynchronous approaches (e-mail, messenger services) can be used when seeking to avoid disruption to daily or necessary schedules of HCPs - Leveraging the use of familiar and straightforward technologies may lead to better engagement by patients and carers than newer technologies requiring adaptation - Telemedicine services could be explored, but must prioritise and seek to retain humanness and face-to-face contact with its use
Potential of DTs	- DT is perceived as a way of making HCPs work more efficiently and include benefits for data storage and security - Remote monitoring (e.g., recording vital signs and reporting symptoms via a mobile phone) is viewed as a useful way of potentially reducing travel and saving time - The use of DTs provide a mode that could support continuous access to services, including psychosocial care, information and services, and support continued engagement for people with limited mobility - There may be opportunities to enhance social connectedness, make new contacts, and potentially reduce isolation when using social media technologies
Reservation and concerns relating to the use of DTs	- There are concerns that misunderstandings could occur where DTs are used without face-to-face, physical communication - Without a clearly articulated reason for new or existing technologies being used by health services, patient and carer uptake may be adversely affected and disinterest may arise - Deviation from familiar, routine modes of communication may lead to disengagement by patients and their family caregivers - The loss of personal contact (i.e., in person and face-to-face contact) may be viewed as impersonal and lack closeness and familiarity - A lack of skills and familiarity with DTs may reduce patient and their caregivers’ willingness to engage or use them - The availability and reliability of network coverage has a strong influence on engagement with DTs for healthcare - Access to quality care may reduce the perceived need for digital transformation of care

Our research builds on earlier work detailing HCP perspectives of DTs [8]. In this study we addressed a critical gap in the evidence base; patient and family caregiver perspectives, which have been underexplored in the context of Germany. Patients and family caregivers are key stakeholders that can influence the uptake and adoption of DT for palliative care [17]. Across the programme of work, there has been alignment between HCP and patient and caregiver perspectives. Both perceive DTs as beneficial to multi-professional collaboration and organisation in the context of palliative care. As demonstrated in findings from the German oVID Project, DTs facilitate intercollegiate collaboration and knowledge exchange among HCPs, enhancing the effectiveness of palliative care [27]. We emphasise the relevance of research into the further development of digital work systems for palliative care [28]. Patient and caregiver preferences elicited from study participants align with the social cognitive theory, an integrated model that highlights the role of personal, environmental and social support as important mediators of behavior [29]. This includes participants’ prior behaviour and familiarity with existing technologies, their perceptions of the value of DT, and preferences for maintenance of face-to-face contact with professionals alongside any DT approach. Moreover, understanding patient and family perspectives on telepalliative care is crucial for its effective implementation, as evidenced by a systematic integrative review [30]. Incorporating patient experiences in the use of telehealth technologies is crucial to ensure that DTs in palliative care remain genuinely patient-centered. The study by Steindal et al. 2020 [31] illustrates how valuable direct feedback from patients can be in identifying areas for technological improvement and enhancing the effectiveness of telehealth. Patient experiences not only reveal how users perceive and utilize the technology but also how it impacts the quality of care. This underscores the necessity for developments in DTs to always consider the needs and preferences of end-users to ensure high acceptance and satisfaction. Such insights are particularly valuable in ensuring that technology does not undermine the human dimension of care but supports and complements it [31]. Further research exploring perspectives through the lens of social cognitive theory may help to develop clearer links between psychosocial factors influencing patient and caregiver willingness to use DT as part of palliative care delivery.

Any DT proposed to a patient may have to be accompanied by a clear case for it use and intended benefit, as it balances any potential technology benefit with the potential loss of highly-valued humanness and personal connectivity with a HCP. Furthermore, patients and family caregivers reported primarily on conventional technologies (i.e. landline phones). The recent enactment of the Act to Accelerate the Digitalisation of the Healthcare System in Germany has specifically mandated the expansion of digital therapeutics (DiGA) and the implementation of electronic patient records (ePA) [19]. These, as well as many other technologies that would in principle be exploitable in palliative care in Germany [32], were not mentioned by our study participants. From this, we draw that there is an implementation gap between what is politically and practically possible in the context of routine palliative care in Germany. This, in turn, highlights the need for data to determine current levels of use of advanced DTs alongside further research to ensure technologies are optimally implemented in line with the preferences of patients and their caregivers [33–35].

### Strengths and limitations

To our knowledge, this is the first exploratory study on patient and caregiver perspectives and preferences for DT use in routine palliative care in Germany. The qualitative study design allowed for an in-depth description of participants’ perceptions of the actual use of DT in day-to-day palliative care delivery. The sample of our study was reflective of patients and family caregivers across multiple settings of palliative care in the region in which data collection took place. A multi-disciplinary team contributed to the analysis and interpretation of data which allowed multiple perspectives and reflections of the interview content. However, limitations to the study exist. The sample included only participants from one federal state in Germany with potentially limited availability of DT and infrastructure. There may have been bias introduced through HCPs who acted as gatekeepers in the recruitment of participants. Gatekeepers may have primarily included people to whom they attributed an interest in DTs or selective in which patients are deemed suited to the study. A comprehensive quantitative survey on the perspectives of patients and caregivers on digitalisation in palliative care is necessary for future research to further explore our qualitative findings, especially concerning the acceptance of DTs for patients and caregivers and potential differences in experiences and needs based on geographic location.

The heavy inclusion of outpatient service users may have skewed the results towards the experiences and needs specific to that setting, potentially emphasizing perspectives and challenges unique to outpatient palliative care. Consequently, our findings might not fully encapsulate the breadth of experiences and issues faced by those in more intensive palliative care environments, such as hospice or inpatient services.

This could also encompass data on symptom burden and socio-economic factors to evaluate which DTs are suitable for specific patients and caregivers, and at what stages of palliative care they should be applied. Another limitation of our study was the lack of patient and public participation in the development of our interview guide and also the fact that the member check in the data analysis was only performed with one participant This decision was due to several constraints, including limited resources and the challenges imposed by the SARS-CoV-2 pandemic, which affected our ability to conduct face-to-face consultations and engage a wider range of stakeholders. The lack of direct input from patients and the public may have impacted the scope and relevance of our topic guide, potentially limiting the diversity of perspectives and depth of insights gained in the interviews.

Our study recognizes regional disparities in the availability of DT across Germany, which could influence the outcomes. These disparities are primarily due to infrastructural differences, where broadband internet access is substantially less developed in rural areas compared to urban centers. Moreover, financial limitations and the prioritization within healthcare budgets can restrict the widespread adoption and effective implementation of advanced digital tools in healthcare settings. Such variability in DT availability can markedly affect the effectiveness of digital health solutions, potentially skewing our study results and limiting their applicability across different regions. Furthermore, the age and technological literacy of participants could impact their perceptions and usage of DT. Given that our study predominantly involved an older population, there may be a variance in the levels of technological literacy, which can influence participants’ confidence in and acceptance of digital healthcare solutions. This generational gap in tech-savviness is a crucial factor and could potentially introduce a bias in their attitudes towards DT.

## Conclusion

The study derives much-needed insights into patient and caregiver perspectives on the use of DT for palliative care in Germany. We elicited and present crucial user requirements that can inform the consideration of technology use within palliative care delivery. The study underscores the need for future research to determine the elements that may influence the uptake and implementation of DTs. As DTs continue to grow in scope and use in palliative care, continued and ongoing user engagement is essential to optimise their adoption and ensure they benefit patients and their caregivers.

### Electronic supplementary material

Below is the link to the electronic supplementary material.

Supplementary Material 1

Supplementary Material 2

## Data Availability

All data relevant to the study are included in the article or uploaded as supplementary material. For further questions regarding the reuse of data, please contact the corresponding author (felix.muehlensiepen@mhb-fontane.de).

## Abbreviations

DT: Digital Technology
HCP: Health Care Professionals
SOPC: Specialised Outpatient Palliative Care
