# Supplementary material for: “Let’s put it this way: you can’t really live without it” - digital technologies in routine palliative care delivery: an explorative qualitative study with patients and their family caregivers in Germany
Source: BMC Health Serv Res. 2024 Jun 3;24:702. doi: 10.1186/s12913-024-11150-5 (PMC11149286; doi:10.1186/s12913-024-11150-5)
Supplement: Supplementary file 1 — Supplementary Material 1 [file 12913_2024_11150_MOESM1_ESM.docx]

**DigiPall – Interview Guide**

| **Key question/ narrative impulse** | **Check aspects** | **Concretizing questions** | **Maintenance and control issues** |
| --- | --- | --- | --- |
| Could you please introduce yourself briefly? | **Warm-up: Introduction/ description of everyday life**  Underlying disease, duration of treatment  Care setting:   - outpatient/inpatient - SAPV, AAPV, palliative care unit, hospice, etc. | How long have you been receiving palliative care?  What does a typical day look like for you?  How do you usually communicate with caregivers? | Can you tell us more about this?  And then?  What was it like for you?  How do you see it?  Can you please go into this in more detail?  Could you please give an example?  What do you mean in concrete terms? |
| The study is about the use of digital technologies in palliative care. What do you think when you hear that? | **Attitude towards digitalization**  Everyday life/ use  Personal attitude towards DT  Data protection! | What is digitalization for you?  What is your general opinion on digitalization? |  |
| "Digital technology" is a collective term for communication applications such as computers, cell phones, smartphones, etc.  **Do you use these devices?** | **Everyday life & DT**  **Attitude towards DT as opposed to private use**    **Status Quo**  Patient/relatives communication  Telephone  Cell phone / Smartphone  Computer / Notebook  Documentation | What forms of DT do you use as part of your treatment?  What forms of DT do you use privately outside of your treatment?  Does your private usage behavior differ from your usage behavior during treatment? If yes, why? What is different?  Why should or should not DT be used in palliative care?  How do you usually communicate when   - Do you have questions for caregivers? - Do you have questions for doctors? - Do you have acute problems? - Do you have a concern during times when nurses or doctors are not usually working?   *For example, in nocturnal pain intervention*  When do you use the landline phone or the cell phone or smartphone as part of your treatment?   - *Calls* - *WhatsApp/ SMS* - *Emails* - *Apps* - *As a source of information: medication, painkillers*   Which devices do you use to communicate with other care providers? *E.g. pharmacy or medical aid provider:in*  What determines which medium you use to communicate?  What role does the computer play in your treatment?   - *Emails* - *Video consultation* - *Monitoring/documentation e.g. of pain, medication; shared documentation* | Can you tell us more about this?  And then?  What was it like for you?  How do you see it?  Can you please go into this in more detail?  Could you please give an example?  What do you mean in concrete terms?  Can you tell us more about this?  And then?  What was it like for you?  How do you see it?  Can you please go into this in more detail?  Could you please give an example?  What do you mean in concrete terms? |
| We have now talked about various devices and communication applications and your everyday working life. Let's look at this in general terms:  **Where and in which areas can digital technology support your treatment? Please describe.** | **Potentials/ opportunities & risks**  -Acceptance  - Potentials  - Risks  - Boundaries  - Users (practitioners, patients, family members and relatives)  - Effects | What opportunities or risks do you see in general?  What opportunities and risks of DT do you see in the following areas when it comes to palliative care?   - *In the organization/ appointment management* - *Diagnostics* - *Treatment* - *Monitoring* - *Medication control* - *Social component* - *Communication (with relatives, if inpatient)*   What problems did you have when using DT?   - *Data protection* - *Connection problems/network coverage* - *Handling*   What limits do you see in the implementation and use of DT in palliative care?  Has Covid-19 changed anything? | Can you tell us more about this?  And then?  What was it like for you?  How do you see it?  Can you please go into this in more detail?  Could you please give an example?  What do you mean in concrete terms? |
|  | **Acknowledgement & farewell** |  |  |

**Socio-demographic data:**

- Age
- Gender
- Care structure (SAPV, palliative care unit, hospice, GP practice, etc.)
- Diagnosis
